# Correlation of triglyceride to high-density lipoprotein cholesterol ratio with nonalcoholic fatty liver disease among the non-obese Chinese population with normal blood lipid levels: a retrospective cohort research

**DOI:** 10.1186/s12944-019-1104-6

**Published:** 2019-08-09

**Authors:** Zekai Chen, Hailun Qin, Shaobin Qiu, Guanzhi Chen, Youren Chen

**Affiliations:** 1 Shantou University Medical College, Shantou, Guangdong China; 2 Department of Cardiology, Second Affiliated Hospital of Shantou University Medical College, Shantou, Guangdong China; 3 Department of Rehabilitation Medicine, Institute of Geriatric Medicine, Guangdong Academy of Medical Sciences, Guangdong Provincial People’s Hospital, Guangzhou, China; 4 China Medical University, Shenyang, Liaoning China

**Keywords:** TG/HDL-C ratio, Non-obese NAFLD, Chinese

## Abstract

**Background:**

Although nonalcoholic fatty liver disease (NAFLD) is commonly seen in metabolic abnormalities patients, NAFLD is also occurred in the non-obese individuals. The ratio of triglyceride to high-density lipoprotein cholesterol (TG/HDL-C) is considered as a predictive factor of NAFLD. However, it is still difficult to confirm the correlation of TG/HDL-C ratio with NAFLD among non-obese Chinese people with normal blood lipid levels. In our study, it is aimed to analyze the correlation of TG/HDL-C ratio with NAFLD among non-obese Chinese population without dyslipidemia.

**Methods:**

In the retrospective cohort study, 9838 non-obese subjects who were free of NAFLD were enrolled. NAFLD was diagnosed by ultrasonography.

**Results:**

During the median follow-up period of 2.9 years, cumulative incidence of NAFLD in non-obesity individuals was 8.69% among the overall population; meanwhile, its incidence was gradually enhanced across the quartiles of TG/HDL-C ratio (0.61, 1.28, 2.55 and 4.25% respectively). Then the multivariate factors were adjusted. The multivariate cox regression analysis results showed that the hazard ratio of NAFLD in higher quartiles (Q2-Q4) was 2.10 (1.33–3.32), 3.11 (2.03–4.75) and 3.40 (2.24–5.17), respectively. Besides, the area under receiver operator characteristic curve (AUC) of TG/HDL-C ratio in the male was 0.70 (0.68–0.72) and 0.72 (0.70–0.75) in the female. The final values were dramatically larger than the other lipid index.

**Conclusion:**

There is an independent relationship between TG/HDL-C and NAFLD among non-obese Chinese population without dyslipidemia, and TG/HDL-C may be used as a better predictor for NAFLD.

## Introduction

In clinical practice, NAFLD is a pathological syndrome that is reflected by excessive fatty deposition of hepatocyte without alcohol use and other causes of liver diseases [1]. The global prevalence of obesity and metabolic syndrome leads to the rising incidence of NAFLD [2]. NAFLD has become a common chronic liver disease. Consequently, a quarter of the global population has been affected [3, 4]. What’s more, the morbidity of NAFLD may dramatically enhance the risks of chronic kidney disease, T2DM and cardiovascular diseases [5–7].

Dyslipidemia is a well-documented influence factor of NAFLD, which can be reflected by the increased total cholesterol (TC) and triglyceride (TG) or declined HDL-C levels and predominance of small dense low-density lipoprotein (sd-LDL) particles [8, 9]. Recently, TG/HDL-C is supposed to be associated with incident NAFLD [10]. TG/HDL-C ratio is a predictive indicator for insulin resistance (IR), T2DM, cardiovascular disease and hypertension [11–14].

Obesity is another major influence factor of NAFLD [15]. If a patient is suffered from obesity or dyslipidemia, considerable attention shall be paid to prevent NAFLD. However, less attention is paid for the non-obese population with normal blood lipid levels. Sun et al. reported that the morbidity of NAFLD is 13.9% among 183,903 non-obese individuals with normal LDL-C levels in China [16]. In order to prevent and manage NAFLD, more attention shall be paid on NAFLD progression among the non-obese people with normal blood lipid levels.

In our paper, it is aimed to study the correlation of TG/HDL-C ratio with NAFLD among the non-obese people with normal blood lipid levels.

## Methods

### Study design & study population

The information about study population was obtained from public dataset offered by Sun et al [16, 17] The research ethics were not required, which had been authorized in the former study according to public policy statements of the dataset. In the cohort study, 16,173 non-obese people who were free of NAFLD were initially enrolled. Exclusion criteria at baseline included: 1) the subjects who had incomplete clinical data and lost to follow; 2) body mass index (BMI) value (≥25 kg/m2); 3) alcohol abuse (> 140 g/w in the male and > 70 g/w in the female); 4) medical history, including NAFLD at the baseline, autoimmune hepatitis, viral hepatitis and known origins of chronic liver disease; 5) dyslipidemia (TC > 5.2 mmol/L, TG > 1.7 mmol/L, LDL-C > 3.12 mmol/L, HDL-C < 1.03 mmol/L); 6) oral medication of anti-hypertensive agents, lipid-lowing drugs or anti-diabetic drugs. Eventually, 9838 subjects (5057 male cases, 51.4%) were asked for an analytical investigation. Selection procedure of study population was specifically revealed in the former research [16].

### Data acquisition

As it was described in the former research, the trained staff would deliver the standardized spreadsheet to collect the general information, such as age, height, gender, systolic blood pressure (SBP), weight, diastolic blood pressure (DBP), medical history, etc. BMI was calculated as weight/height2 at kg/m2. Under fasting conditions, laboratory parameters were recorded by professional investigators.

### Definition

Diagnosis criteria of NAFLD conformed to Chinese Society of Hepatology (2010). In general, NAFLD was diagnosed with two of three abnormalities at least, namely, diffuse hyperechogenicity of the liver with respect to the spleen and kidney, attenuated ultrasound beam, and poorly visualized intrahepatic architectural details [18]. TG/HDL-C ratio was calculated as plasma TG levels (mmol/L) divided by HDL-C levels (mmol/L).

### Statistical analysis

The subjects were assigned to four groups based on the quartiles of TG/HDL-C ratio: Q1 (≤0.46), Q2 (0.47–0.63), Q3 (0.64–0.86) and Q4 (≥0.87). The baseline characteristics were described and compared. For the normal distribution, continuous variables were expressed as median (quartile) and for abnormal distribution, they were expressed as mean ± standard deviation (SD). For abnormal distribution and normal distribution, group comparison for continuous variables was conducted through a non-parametric test and one-way ANOVA. Chi-squared test was used to compare the categorical variables. The incidence rate of predefined outcome was counted by person-years incidence and cumulative incidence. The cumulative incidence was compared by log-rank test. In order to analyze the correlation of TG/HDL-C with NAFLD, the cox proportional hazards model was applied to evaluate the risks of NAFLD while obtaining *P* values and Hazard ratio (HRs) with 95% confidence intervals (CIs). ROC curve analysis was used to investigate the possibilities of TG/HDL-C and other lipid index for diagnosis of NAFLD. *P* < 0.05 (two-tailed) showed statistical significance. SPSS System version 23.0 was used for statistical analysis (SPSS Inc., Chicago, IL, USA).

## Results

### Demographic and clinical characteristic of the subjects at baseline

In this study, 9838 subjects without NAFLD at baseline were covered. The subjects had a mean age of 42.5 years old; the male cases accounted for 51.4% of the overall subjects. The average value of BMI was 21.1 kg/m2. According to TG/HDL-C ratio, the subjects were grouped. In addition, the baseline biochemical and clinical features were depicted (Table 1). Compared with subjects of higher TG/HDL-C ratio, the subjects of lower TG/HDL-C ratio had higher clinical indexes like BMI, age, DBP, SBP, fasting plasma glucose (FPG), albumin, alanine aminotransferase (ALT), alkaline phosphatase (ALP), aspartate transaminase (AST), gamma glutamyl transpeptidase (GGT), creatinine, uric acid, TG, total cholesterol (TC) and LDL-C as well as the number of the males. In Table 1, the results showed that individuals with higher TG/HDL-C ratio had lower HDL-C and direct bilirubin levels by comparison with individuals of lower TG/HDL-C ratio (*P* < 0.001).

**Table 1:** Baseline demographic and clinical characteristics of the participants

Characteristics	Quartiles of TG/HDL-C Ratio				*P* value
	Q1 (≤0.46)	Q2 (0.47–0.63)	Q3 (0.64–0.86)	Q4 (≥0.87)	
Age (years)	41.7 ± 14.3	42.5 ± 14.8	42.7 ± 14.8	43.0 ± 14.9	0.010
Gender, male/female (n)	1188/1340	1247/1172	1278/1184	1344/1085	< 0.001
Body mass index (kg/m^2^)	20.3 ± 1.9	20.8 ± 2.0	21.3 ± 2.0	21.8 ± 2.0	< 0.001
Systolic blood pressure (mm Hg)	114.0 ± 14.8	116.6 ± 15.9	119.8 ± 16.5	122.0 ± 16.0	< 0.001
Diastolic blood pressure (mm Hg)	69.0 ± 9.3	70.5 ± 9.9	72.1 ± 10.0	73.6 ± 10.1	< 0.001
Fasting plasma glucose (mmol/L)	4.94 ± 0.59	5.05 ± 0.63	5.11 ± 0.64	5.20 ± 0.89	< 0.001
Albumin (U/L)	44.1 ± 2.7	44.2 ± 2.8	44.3 ± 2.7	44.5 ± 2.8	< 0.001
Alanine aminotransferase (U/L)	16.6 ± 14.2	17.8 ± 21.0	18.9 ± 12.8	20.6 ± 14.5	< 0.001
Aspartate aminotransferase (U/L)	21.5 ± 8.6	22.1 ± 10.4	22.4 ± 8.1	22.8 ± 8.8	< 0.001
Alkaline phosphatase (U/L)	63.7 ± 21.0	68.0 ± 20.3	71.8 ± 23.6	75.1 ± 22.9	< 0.001
Gamma glutamyl transpeptidase (U/L)	20.6 ± 20.2	22.3 ± 14.8	27.1 ± 27.4	29.3 ± 24.9	< 0.001
Creatinine (mmol/L)	70.7 ± 15.5	75.2 ± 22.9	79.3 ± 32.1	83.3 ± 26.5	< 0.001
Uric acid (μmol/L)	233.4 ± 68.3	253.4 ± 77.1	274.8 ± 79.3	296.1 ± 80.2	< 0.001
Direct bilirubin (μmol/L)	2.40 (1.60–2.90)	2.38 (1.60–2.80)	2.31 (1.50–2.80)	2.31 (1.50–2.80)	0.003
Total cholesterol (mmol/L)	4.31 ± 0.53	4.31 ± 0.54	4.34 ± 0.55	4.40 ± 0.50	< 0.001
Triglyceride (mmol/L)	0.64 (0.55–0.72)	0.85 (0.75–0.93)	1.05 (0.93–1.16)	1.36 (1.22–1.50)	< 0.001
HDL cholesterol (mmol/L)	1.78 ± 0.29	1.57 ± 0.24	1.43 ± 0.22	1.26 ± 0.16	< 0.001
LDL cholesterol (mmol/L)	1.96 ± 0.39	2.08 ± 0.40	2.16 ± 0.42	2.27 ± 0.39	< 0.001
TG/HDL-C ratio	0.36 ± 0.07	0.54 ± 0.05	0.73 ± 0.07	1.08 ± 0.17	< 0.001

Data are described by mean ± standard deviation or median (quartile)

*Abbreviation:* HDL = high-density lipoprotein; LDL = low-density lipoprotein; TG = triglyceride

### Incidence rate of NAFLD in non-obese patients

During the median follow-up period of 2.9 years, there were 855 non-obesity individuals with NAFLD (Table 2). Incidence rate of total incident NAFLD was 345.2 per 10,000 person-years. Across the quartiles 1, 2, 3, and 4 of TG/HDL ratio, the cumulative incidence of NAFLD among non-obesity individuals was increased from (0.61(0.46–0.76), 1.28(1.06–1.50), 2.55(2.24–2.86) to 4.25(3.85–4.65) (Table 2).

**Table 2:** Incidence rate of NAFLD stratified by TG/HDL-C ratio

Group	Number	No. of NAFLD	Cumulative incidence (95% CI)	Per10,000 person-years
Total	8983	855	8.69 (8.13–9.25)	345.2
Q1 (≤0.46)	2528	60	0.61 (0.46–0.76)	82.8
Q2 (0.47–0.63)	2419	126	1.28 (1.06–1.50)	189.5
Q3 (0.64–0.86)	2462	251	2.55 (2.24–2.86)	377.1
Q4 (≥0.87)	2429	418	4.25 (3.85–4.65)	635.0
P value for log-rank test			< 0.001	

*Abbreviation:* NAFLD = non-alcoholic fatty liver disease; TG = triglyceride; HDL = high-density lipoprotein; CI = confidence interval

### Correlation of TG/HDL-C with NAFLD in non-obese people

The analysis results of the multivariate cox proportional hazard regression were displayed in Table 3. In Model 1 and Model 2 (the age and gender were adjusted), higher TG/HDL-C was statistically associated with NAFLD in non-obese people. While adjusting BMI, FPG, albumin, SBP, DBP, ALT, AST, BUN, Cr, uric acid and direct bilirubin, the hazard ratio (HR) for non-obese NAFLD patients was gradually enhanced across the quartiles of TG/HDL-C ratio. Compared with Q1, HR for non-obese NAFLD patients was 2.10(1.33–3.32), 3.11(2.03–4.75) and 3.40(2.24–5.17), respectively.

**Table 3:** Hazard ratio (95% CI) of NAFLD stratified by TG/HDL-C ratio

Group	Model 1	Model 2	Model 3
Q1 (≤0.46)	1	1	1
Q2 (0.47–0.63)	2.28 (1.68–3.10)	2.27 (1.67–3.08)	2.13 (1.35–3.36)
Q3 (0.64–0.86)	4.54 (3.42–6.01)	4.50 (3.40–5.97)	3.11 (2.04–4.76)
Q4 (≥0.87)	7.57 (5.77–9.92)	7.48 (5.71–9.81)	3.44 (2.27–5.23)
P value	< 0.001	< 0.001	< 0.001

*Note:* Model 1 is univariate analysis; Model 2 is adjusted for age and sex; Model 3 further adjusted for body mass index, fasting plasma glucose, albumin, systolic blood pressure, diastolic blood pressure, alanine aminotransferase, aspartate aminotransferase, creatinine, uric acid, direct bilirubin, alkaline phosphatase, gamma glutamyl transpeptidase

*Abbreviations:* NAFLD = non-alcoholic fatty liver disease; TG = triglyceride; HDL = high-density lipoprotein; CI = confidence interval

### ROC analysis of TG/HDL-C and risks for NAFLD in non-obese people

**Fig. 1:**
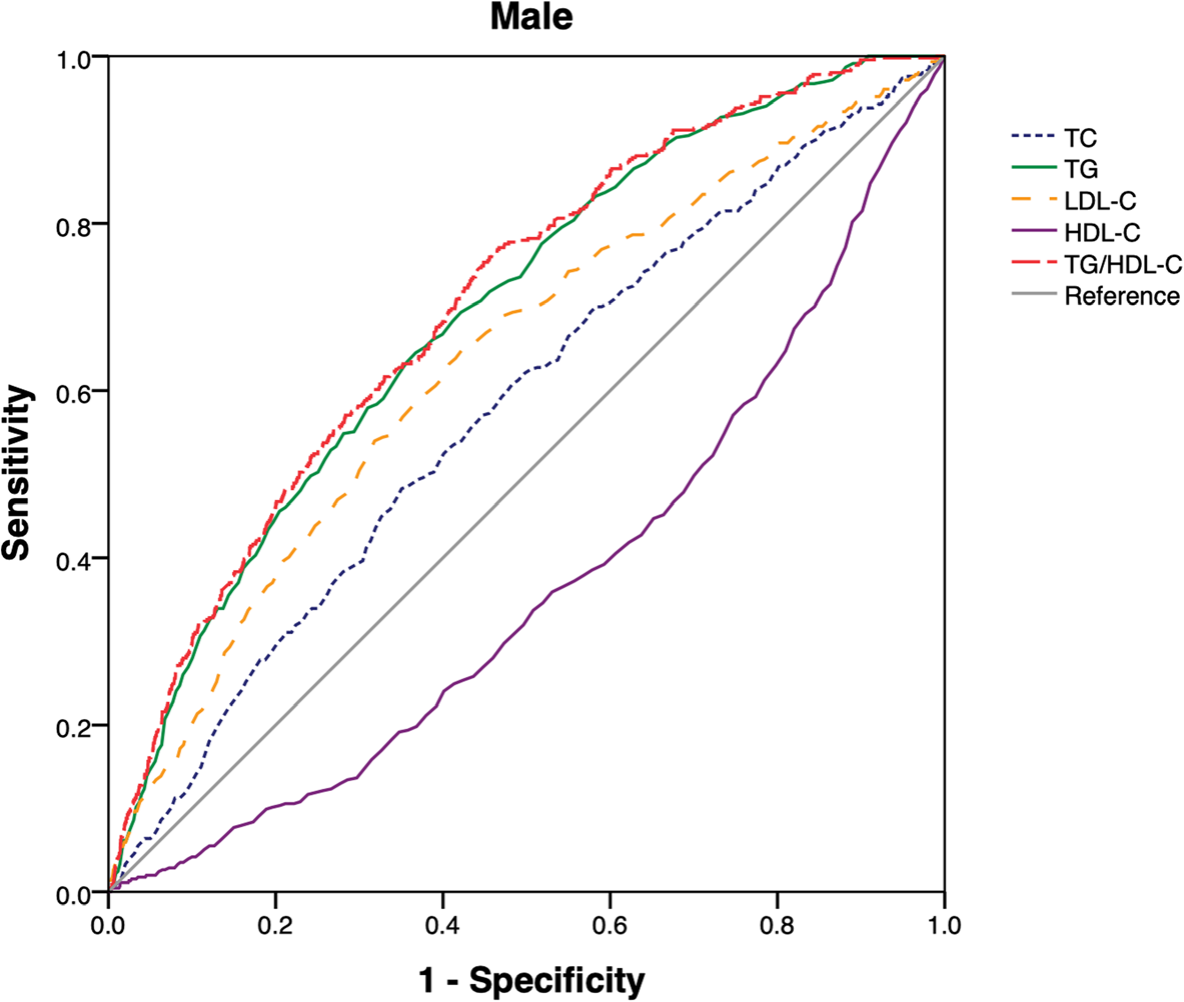
ROC curves of TG/HDL-C, TC, TG, LDL-C and HDL-C were presented in male

**Fig. 2:**
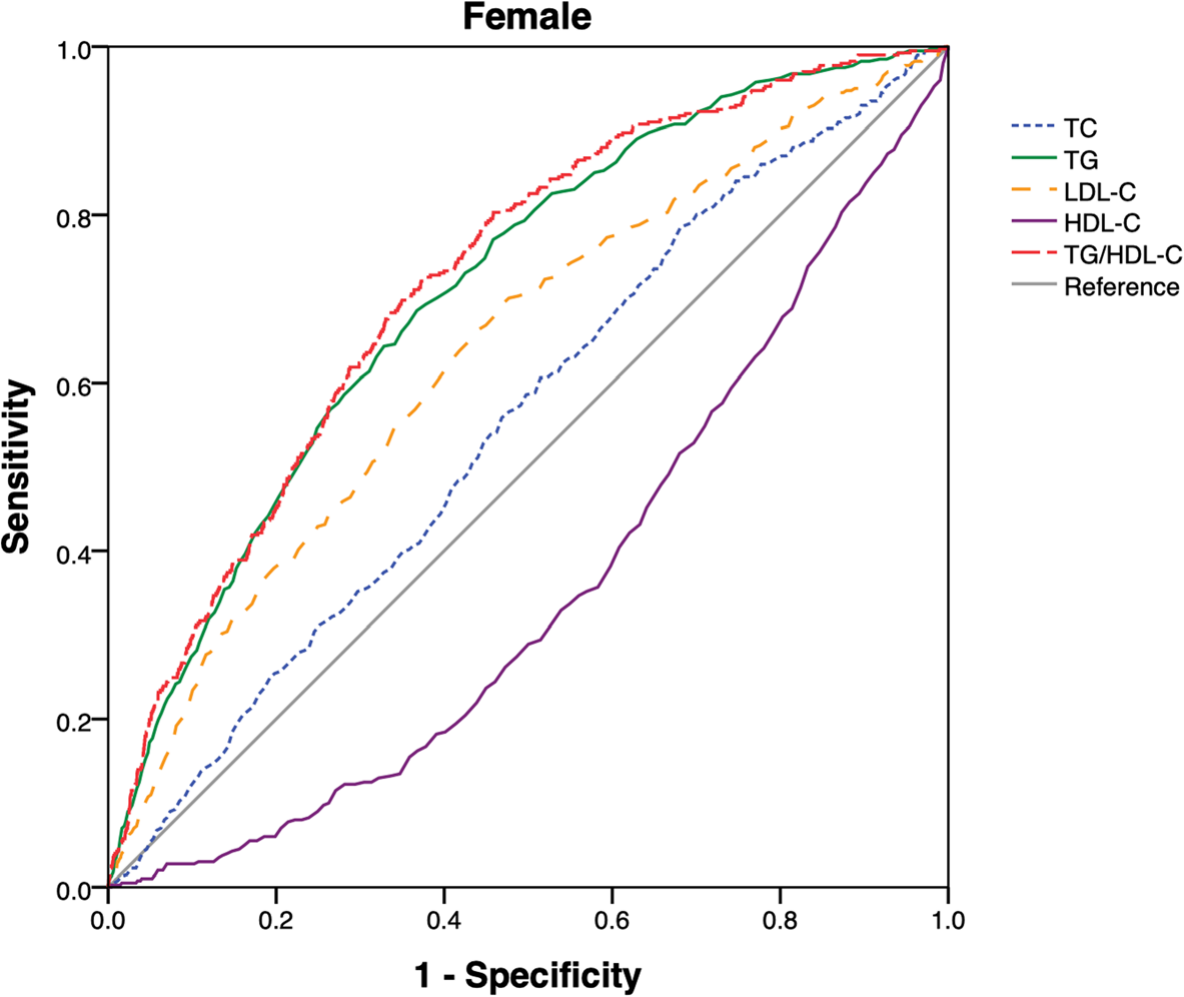
ROC curves of TG/HDL-C, TC, TG, LDL-C and HDL-C were presented in female. *Abbreviation:* TC = cholesterol; TG = triglyceride; LDL-C = low-density lipoprotein cholesterol; HDL-C = high-density lipoprotein cholesterol

ROC values of TG/HDL-C, HDL-C, LDL-C, TG and TC were shown in Fig. 1 and Fig. 2. For the male or the female, AUC value of TC/HDL-C ratio was larger than that of HDL-C, LDL-C, TC and TG. The results indicated that TC/HDL-C had better predictive effects than other lipid index. Furthermore, the optimal cut-off point of TC/HDL-C ratio for non-obese NAFLD patients was 0.65 in the male and 0.69 in the female.

## Discussion

As far as we know, this is the first retrospective cohort study to demonstrate the prediction effects of TG/HDL-C for detection of NAFLD among non-obese Chinese people with normal blood lipid levels. The research findings show that the subjects with increased TG/HDL-C may have higher risks and cumulative incidence of NAFLD among non-obesity individuals with normal lipid levels. According to AUC of TG/HDL-C for non-obese NAFLD patients, we have found that compared with other lipid index, TG/HDL-C can better predict NAFLD among non-obese population. Our study might be helpful to identify the high-risk subjects for the specific prevention measures.

With the economic boom and quick adoption of western lifestyle in the Asian-Pacific region, NAFLD is prevalent in non-obesity individuals [19]. The proportion of the non-obesity individuals with NAFLD is ranged between 16% in Italy, 17% in the US, 19% in Hong Kong, 27% in Korea and 75% in India [20–24]. After the follow-up periods of five years, the cumulative incidence of NAFLD reaches up to 8.69% in the non-obese Chinese population with normal blood lipid levels. Nonalcoholic steatohepatitis (NASH) is a severe form of NAFLD. The prevalence of NAFLD in adults is about 20–30%, of which nearly 10–25% patients with NAFLD can progress to NASH [25]. And approximately 21–26% of patients with NASH progress to cirrhosis within 8.2 years [26]. From 2003 to 2014, the number of liver transplantation secondary to NASH increased 162%, and NASH-related cirrhosis has assumed prominence as being currently the second leading indication for liver transplantation in the USA [27]. Therefore, it is necessary to find out an accurate predictive marker for non-obese NAFLD population with normal blood lipid levels.

In this paper, we have found that owing to elevated TG/HDL-C, the risks of NAFLD in other three groups are statistically higher than those of control group. Risks of NAFLD in the fourth quintile are as 3.4 times high as those in the first quintile. With this dose-response relationship, ROC analysis results show TG/HDL-C ratio has higher predictive value than other lipid index. AUC of TG/HDL-C ratio in the male is 0.70(0.68–0.73) and 0.72 (0.70–0.75) in the female. Fan et al. reported the similar results. Compared with other lipid index and liver injury markers, AUC of TG/HDL-C ratio in patients with NAFLD is 0.79 in the male and 0.85 in the female [10]. In our study, the optimal cut-off point of TG/HDL-C ratio in non-obese NAFLD population with normal blood lipid levels is 0.65 in the male and 0.69 in the female. Fukuda et al. stated that the optimal cut-off point of TG/HDL-C ratio in incident fatty liver patients is 0.64 in the female and 0.88 in the male [28].

There are some possible mechanisms about the correlation of TG/HDL-C ratio with NAFLD in non-obesity individuals. Firstly, IR has a close correlation with NAFLD among non-obese people [29]; TG/HDL-C could be seen as the independent predictive factor for IR [30]. With high TG levels, free fatty acids (FFAs) are increased with better lipolysis. The elevated FFAs levels can bring about the deterioration of insulin sensitivity; the induction of tissue oxidative stress will lead to tissue insulin resistance. With the decreased anti-oxidation and anti-inflammation ability, lower HDL-C levels may lead to IR [31, 32]. Therefore, TG/HDL-C may have better predictability for IR and NAFLD in the non-obese people. Secondly, a variant allele (rs738409) of PNPLA3 is increased in non-obesity patients with NAFLD; it could be seen as the independent influence factor of non-obese NAFLD population [33, 34]. Wei et al. showed that among 78.4% of non-obese NAFLD patients of carrying PNPLA3 rs738409 [22]. According to Dallas Heart Study, PNPLA3 rs738409 is strongly associated with increased hepatic TG levels and hepatic inflammation [35]. Interestingly, clinical epidemiological data shows that even if metabolic abnormalities like obesity, dyslipidemia or T2DM are absent, NAFLD may be seen in the subjects with variant PNPLA3 [36, 37]. Therefore, PNPLA3 rs738409 is significant to assess the correlation of TG/HDL-C ratio with NAFLD among non-obese population of normal blood lipid levels.

This research has some advantages, for instance, long-term follow-up visit, retrospective design and large sample size. However, there are still some limitations. First of all, the ultrasonic examination is used to diagnose NAFLD, but the severity of NAFLD cannot be defined. At the same time, the non-invasive and economical approach has been clinically applied for epidemiological studies [38]. Secondly, our primary study design cannot enable the examination of insulin contents and IR that could have a close association with NAFLD among the non-obese individuals. Nevertheless, the main purpose is to probe into the predictive value of TG/HDL-C for non-obese NAFLD population. Thirdly, questionnaire is not delivered to collect the information, such as their medical history, lifestyle, eating behaviours, frequency of exercise, etc. Through the adjustment of multivariate factors, some important variables are missing.

In conclusion, NAFLD is prevalently occurred among non-obese Chinese people with normal lipid levels. TG/HDL-C is the independent predictive risk factor for NAFLD. Therefore, the non-obese people with normal blood lipid levels and higher TG/HDL-C ratio shall be particularly concerned. Our research findings may provide basis for policy-makers to implement the schemes towards high-risk non-obese people with normal lipid levels.

## Conclusion

There is an independent relationship between TG/HDL-C and NAFLD among non-obese Chinese population without dyslipidemia, which may be used as a better predictor for NAFLD.

## Data Availability

The datasets analyzed during the current study are available from the corresponding author on reasonable request.

## References

[1] Neuschwander-Tetri BA, Caldwell SH (2003). Nonalcoholic steatohepatitis: summary of an AASLD single topic conference. Hepatology.

[2] Khashab MA, Liangpunsakul S, Chalasani N (2008). Nonalcoholic fatty liver disease as a component of the metabolic syndrome. Curr Gastroenterol Rep.

[3] Lonardo A, Bellentani S, Argo CK (2015). Epidemiological modifiers of non-alcoholic fatty liver disease: focus on high-risk groups. Dig Liver Dis.

[4] Younossi ZM, Koenig AB, Abdelatif D (2016). Global epidemiology of nonalcoholic fatty liver disease-meta-analytic assessment of prevalence, incidence, and outcomes. Hepatology.

[5] Targher G, Day CP, Bonora E (2010). Risk of cardiovascular disease in patients with nonalcoholic fatty liver disease. N Engl J Med.

[6] Sung KC, Kim SH (2011). Interrelationship between fatty liver and insulin resistance in the development of type 2 diabetes. J Clin Endocrinol Metab.

[7] Targher G, Bertolini L, Rodella S (2008). Non-alcoholic fatty liver disease is independently associated with an increased prevalence of chronic kidney disease and proliferative/laser-treated retinopathy in type 2 diabetic patients. Diabetologia.

[8] Yang S, Du Y, Li X (2017). Triglyceride to high-density lipoprotein cholesterol ratio and cardiovascular events in diabetics with coronary artery disease. Am J Med Sci.

[9] Wu L, Parhofer KG (2014). Diabetic dyslipidemia. Metabolism.

[10] Fan N, Peng L, Xia Z et al. Triglycerides to high-density lipoprotein cholesterol ratio as a surrogate for nonalcoholic fatty liver disease: A cross-sectional study. Lipids Health Dis. 2019;18(1). 10.1186/s12944-019-0986-7. 10.1186/s12944-019-0986-7 PMC6359827 30711017

[11] Mclaughlin T, Reaven G, Abbasi F (2005). Is there a simple way to identify insulin-resistant individuals at increased risk of cardiovascular disease?. Am J Cardiol.

[12] He S, Wang S, Chen XP (2012). Higher ratio of triglyceride to high-density lipoprotein cholesterol may predispose to diabetes mellitus: 15-year prospective study in a general population. Metab Clin Exp.

[13] Turak O, Afar B, Ozcan F (2016). The role of plasma triglyceride/high-density lipoprotein cholesterol ratio to predict new cardiovascular events in essential hypertensive patients. J Clin Hypertens.

[14] Miller M, Stone NJ, Ballantyne C (2011). Triglycerides and cardiovascular disease a scientific statement from the American Heart Association. Circulation.

[15] Fabbrini E, Sullivan S, Klein S (2010). Obesity and nonalcoholic fatty liver disease: biochemical, metabolic, and clinical implications. Hepatology.

[16] Sun DQ, Wu SJ, Liu WY (2016). Association of low-density lipoprotein cholesterol within the normal range and NAFLD in the non-obese Chinese population: a cross-sectional and longitudinal study. BMJ Open.

[17] Sun DQ, Wu SJ, Liu WY, et al. Data from: association of low-density lipoprotein cholesterol within the normal range and NAFLD in the non-obese Chinese population: a cross-sectional and longitudinal study. Dryad Digital Repository. 2016; 10.5061/dryad.1n6c4. 10.1136/bmjopen-2016-013781 PMC5168665 27927668

[18] Zeng MD, Fan JG, Lu LG (2008). Guidelines for the diagnosis and treatment of nonalcoholic fatty liver diseases. J Dig Dis.

[19] Xu C, Yu C, Ma H (2013). Prevalence and risk factors for the development of nonalcoholic fatty liver disease in a nonobese Chinese population: the Zhejiang Zhenhai study. Am J Gastroenterol.

[20] Bellentani S, Saccoccio G, Masutti F (2000). Prevalence of and risk factors for hepatic steatosis in northern Italy. Ann Intern Med.

[21] Browning JD, Szczepaniak LS, Dobbins R (2004). Prevalence of hepatic steatosis in an urban population in the United States: impact of ethnicity. Hepatology.

[22] Wei JL, Leung JC, Loong TC (2015). Prevalence and severity of nonalcoholic fatty liver disease in non-obese patients: a population study using proton-magnetic resonance spectroscopy. Am J Gastroenterol.

[23] Sinn DH, Gwak GY, Park HN (2012). Ultrasonographically detected non-alcoholic fatty liver disease is an independent predictor for identifying patients with insulin resistance in non-obese, non-diabetic middle-aged Asian adults. Am J Gastroenterol.

[24] Das K, Mukherjee PS, Ghosh A (2010). Nonobese population in a developing country has a high prevalence of nonalcoholic fatty liver and significant liver disease. Hepatology.

[25] Ong JP, Elariny H, Collantes R (2005). Predictors of nonalcoholic steatohepatitis and advanced fibrosis in morbidly obese patients. Obes Surg.

[26] Matteoni CA (1999). Nonalcoholic fatty liver disease: a spectrum of clinical and pathological severity. Gastroenterology.

[27] Cholankeril G, Wong RJ, Hu M (2017). Liver transplantation for nonalcoholic steatohepatitis in the US: temporal trends and outcomes. Dig Dis Sci.

[28] Fukuda Y, Hashimoto Y, Hamaguchi M (2016). Triglycerides to high-density lipoprotein cholesterol ratio is an independent predictor of incident fatty liver; a population-based cohort study. Liver Int.

[29] Sinn DH, Gwak GY, Park HN (2012). Ultrasonographically detected nonalcoholic fatty liver disease is an independent predictor for identifying patients with insulin resistance in non-obese, non-diabetic middle-aged Asian adults. Am J Gastroenterol.

[30] GonzálezChávez A, SimentalMendía LE, ElizondoArgueta S (2011). Elevated triglycerides /HDL-cholesterol ratio associated with insulin resistance. Cir Cir.

[31] Lam TK, Carpentier A, Lewis GF (2003). Mechanisms of the free fatty acid–induced increase in hepatic glucose production. Am J Physiol Endocrinol Metab.

[32] Lewis GF, Carpentier A, Adeli K (2002). Disordered fat storage and mobilization in the pathogenesis of insulin resistance and type 2 diabetes. Endocr Rev.

[33] Feldman A, Eder SK, Felder TK (2016). Clinical and metabolic characterization of lean Caucasian subjects with non-alcoholic fatty liver. Am J Gastroenterol.

[34] Krawczyk Marcin, Bantel Heike, Rau Monika, Schattenberg Jörn M., Grünhage Frank, Pathil Anita, Demir Münevver, Kluwe Johannes, Boettler Tobias, Weber Susanne N., Geier Andreas, Lammert Frank (2018). Could inherited predisposition drive non-obese fatty liver disease? Results from German tertiary referral centers. Journal of Human Genetics.

[35] Romeo S, Kozlitina J, Xing C (2008). Genetic variation in PNPLA3 confers susceptibility to nonalcoholic fatty liver disease. Nat Genet.

[36] Speliotes EK, Butler JL, Palmer CD (2010). PNPLA3 variants specifically confer increased risk for histologic nonalcoholic fatty liver disease but not metabolic disease. Hepatology.

[37] Shen J, Wong GL, Chan HL (2014). PNPLA3 gene polymorphism accounts for fatty liver in community subjects without metabolic syndrome. Aliment Pharmacol Therap.

[38] Angulo P (2002). Nonalcoholic fatty liver disease. N Engl J Med.

